# Recent insights: mesenchymal stromal/stem cell therapy for acute respiratory distress syndrome

**DOI:** 10.12688/f1000research.8217.1

**Published:** 2016-06-28

**Authors:** Shahd Horie, John G. Laffey

**Affiliations:** 1 Anaesthesia, School of Medicine, Clinical Sciences Institute, National University of Ireland, Galway, Ireland; 2 Regenerative Medicine Institute, National University of Ireland, Galway, Ireland; 3 Department of Anesthesia, Critical Illness and Injury Research Centre, Keenan Research Centre for Biomedical Science, St Michael’s Hospital, University of Toronto, Toronto, Canada

**Keywords:** ARDS, Acute respiratory distress syndrome, Mesenchymal stromal/stem cells, MSCs

## Abstract

Acute respiratory distress syndrome (ARDS) causes respiratory failure, which is associated with severe inflammation and lung damage and has a high mortality and for which there is no therapy. Mesenchymal stromal/stem cells (MSCs) are adult multi-progenitor cells that can modulate the immune response and enhance repair of damaged tissue and thus may provide a therapeutic option for ARDS. MSCs demonstrate efficacy in diverse *in vivo* models of ARDS, decreasing bacterial pneumonia and ischemia-reperfusion-induced injury while enhancing repair following ventilator-induced lung injury. MSCs reduce the pro-inflammatory response to injury while augmenting the host response to bacterial infection. MSCs appear to exert their effects via multiple mechanisms—some are cell interaction dependent whereas others are paracrine dependent resulting from both soluble secreted products and microvesicles/exosomes derived from the cells. Strategies to further enhance the efficacy of MSCs, such as by overexpressing anti-inflammatory or pro-repair molecules, are also being investigated. Encouragingly, early phase clinical trials of MSCs in patients with ARDS are under way, and experience with these cells in trials for other diseases suggests that the cells are well tolerated. Although considerable translational challenges, such as concerns regarding cell manufacture scale-up and issues regarding cell potency and batch variability, must be overcome, MSCs constitute a highly promising potential therapy for ARDS.

## Introduction

Acute respiratory distress syndrome (ARDS) constitutes a spectrum of increasingly severe acute respiratory failure that may account for 10% of intensive care unit admissions worldwide and continues to have a mortality rate of over 40% ^1^. ARDS can develop in response to multiple predisposing factors, including pneumonia, systemic infection, and major surgery or multiple traumas ^1^. According to the Berlin Definition, the disease can be classified as mild, moderate, or severe, depending on the severity of hypoxemia ^2^. Patients with ARDS have an acute onset of symptoms and signs that include severe dyspnea, chest pain, and cyanosis and bilateral pulmonary infiltrates indicative of edema on chest radiography; these symptoms usually appear within a one-week period from a known clinical insult ^2^.

Inflammation is one of the main drivers of ARDS pathogenesis. Damage to the lung endothelial and epithelial layers is caused by inflammation of the lung parenchyma which is mediated by a predominantly neutrophilic influx into the alveolar space ^3^. This leads to the release of pro-inflammatory cytokines, including interleukin-6 (IL-6), IL-1β, and IL-8 and tumor necrosis factor-alpha (TNF-α) ^3^. This in turn leads to the generation of reactive oxygen species that can impair lung barrier function and increase vascular permeability and finally, where ARDS persists, can cause end-stage fibrosis ^3^. Currently, there are no therapies for this condition, and protective mechanical ventilation and fluid-restrictive strategies are the only supportive treatments that can improve outcome. Mesenchymal stromal/stem cells (MSCs) have attracted considerable interest as a potential therapy for ARDS. In this review, we will discuss early key studies that first demonstrated the potential of MSCs for ARDS and subsequently focus on recent findings and advances published within the last 2 years.

## Rationale for mesenchymal stromal/stem cell therapy for acute respiratory distress syndrome

MSCs are multipotent adult progenitor cells that can be isolated from numerous sources, including bone marrow, umbilical cord, and adipose tissue, and can differentiate into mesenchymal lineage cells such as fat or cartilage tissue ^4^. The interest in MSCs as a possible therapy for ARDS stems largely from their potential to modulate the host immune response to injury and infection and to promote repair following tissue injury. A number of seminal studies showed the therapeutic potential of MSCs for ARDS. Gupta *et al.* demonstrated that MSCs significantly improve survival and reduced lung injury in mice following *Escherichia coli* endotoxin lung injury ^5^. Importantly, MSCs modulated the immune response by inhibiting TNF-α release and by enhancing anti-inflammatory IL-10 secretion ^5^. Mei *et al.* demonstrated that MSCs genetically modified to overexpress angiopoietin 1, an important protein involved in vascular growth and angiogenesis, were more effective than naïve MSC treatment in a mouse model of lipopolysaccharide (LPS)-induced lung injury ^6^. Chang *et al.* demonstrated that human umbilical cord blood-derived MSCs attenuated hyperoxia-induced lung injury in the neonatal rat, a key demonstration of the efficacy of MSCs from other, potentially more plentiful, tissue sources ^7^. Nemeth *et al.* demonstrated the therapeutic potential of MSCs in a murine model of systemic sepsis, and efficacy was mediated via reprogramming of macrophage function ^8^. Of translational significance, Lee *et al.* demonstrated that human MSCs and MSC conditioned medium (MSC-CM) decreased endotoxin-induced injury to the human *ex vivo* perfused lung via mechanisms involving improved alveolar fluid clearance ^9^. Krasnodembskaya *et al.* highlighted the role of MSC-secreted antimicrobial peptides in augmenting the host response to bacterial infection ^10^. Curley *et al.* demonstrated the potential for MSC therapy to repair the lung following ventilation-induced acute lung injury (ALI) ^11^.

The immune-modulating effect of MSCs is increasingly well understood. MSCs promote T regulatory cell expansion, which causes the suppression of the proliferation of effector T cells and dampens the immune response ^12^. Furthermore, MSCs can modify T cells, dendritic cells, and natural killer cells and decrease their release of pro-inflammatory cytokines or increase their release of anti-inflammatory molecules ^12^. MSCs can also cause the release of soluble potent anti-inflammatory mediators such as indoleamine 2,3-dioxygenase (IDO), prostaglandin E _2_ (PGE _2_), and IL-10 ^12, 13^. In terms of their reparative function, MSCs secrete growth factors such as keratinocyte growth factor (KGF) and vascular endothelial growth factor (VEGF) ^14, 15^. Importantly for an acute inflammatory disorder such as ARDS, MSCs do not elicit a significant immune response in the host and thus can be used for allogeneic transplantation ^12^.

## Recent insights regarding mesenchymal stromal/stem cells in preclinical acute respiratory distress syndrome models

MSCs have previously shown promising efficacy in numerous preclinical ARDS models, including pneumonia, ventilator-induced lung injury (VILI), and sepsis. A recent study showed that mouse MSCs significantly improved survival, reduced histologic lung injury, and reduced pulmonary edema in a mouse model of *E. coli*-induced ALI via a mechanism involving reduced oxidant stress ( Table 1) ^16^. Devaney *et al.* also recently demonstrated that human bone marrow (hBM)-derived MSCs reduce injury severity and increase survival in a rat model of *E. coli* pneumonia ( Table 1) ^17^. hBM-MSCs reduced the lung bacterial load following intra-tracheal *E. coli* administration via a mechanism involving enhanced antimicrobial peptide secretion and increased macrophage phagocytosis ^17^. hBM-MSCs decreased alveolar concentrations of IL-6 while increasing concentrations of the anti-inflammatory cytokine IL-10 and increasing KGF ^17^. Other findings of translational significance included delineation of the human MSC dose response curve and the demonstration that cryopreserved cells retain efficacy, but the secretome alone was less effective in the setting of *E. coli* pneumonia ^17^. In a key study, Asmussen *et al.* showed that clinical-grade human MSCs significantly enhanced oxygenation while reducing pulmonary edema without any adverse effects in a large animal (sheep) model of combined smoke inhalation and *Pseudomonas aeruginosa* pneumonia ( Table 1) ^18^. The translational importance of this study is underlined by the fact that these cells are now in early phase clinical trials for ARDS.

**Table 1.** Recent insights into mesenchymal stromal/stem cell efficacy in preclinical models.

Injury setting	Cell therapy	Effects
**Unmodified MSC therapy**		
*Escherichia coli*-induced ALI in mice ^16^	Mouse bone marrow MSCs 5 × 10 ^5^ cells/mL/22g IV	- Increased survival - Decreased lung edema and lung histology score - Decreased oxidative stress injury (decreased myeloperoxidase activity and malondialdehyde levels)
*E. coli* pneumonia in rats ^17^	hBM MSCs 10 and 20 million per kilogram IV	- Increased oxygenation and survival - Decreased lung histology score and bacterial load - Frozen delivery of cells is effective - Increased phagocytosis and LL-37 release *in vitro*
*Pseudomonas aeruginosa* administration and smoke inhalation in sheep ^18^	hBM MSCs 5 and 10 million per kilogram IV	- Increased oxygenation - Decreased lung edema - No adverse effects observed
Repair after ventilator- induced lung injury in rats ^21^	hBM MSCs 1–10 million per kilogram intra-tracheally and IV	- Increased blood oxygenation and lung compliance - Decreased lung edema and histologic injury - Decreased bronchoalveolar lavage CINC-1 and IL-6 - Lowest efficacious dose is 2 million per kilogram
*Ex vivo* lungs under prolonged ischemic conditions ^23^	hBM MSCs 5 million per kilogram IV	Decreased edema in human lungs rejected for transplantation
**Modified MSC therapy**		
Lipopolysaccharide- induced endothelial injury *in vitro* ^24^	MSCs overexpressing ACE2	Enhanced decrease in endothelial damage, edema, and TNF-α and IL-6 concentrations when compared with naïve MSCs
Bleomycin-induced ALI in mice ^25^	Human umbilical cord MSCs overexpressing ACE2	Enhanced decrease in TNF-α, interferon-gamma, transforming growth factor-beta, and IL-1β, IL-2, and IL-6 and enhanced increase of superoxide dismutase and IL-10 when compared with naïve MSCs

Abbreviations: ACE2, angiotensin-converting enzyme 2; ALI, acute lung injury; CINC-1, cytokine-induced neutrophil chemoattractant 1; hBM, human bone marrow; IL, interleukin; IV, intravenously; MSC, mesenchymal stromal/stem cell; TNF-α, tumor necrosis factor-alpha.

Previous studies observed MSCs to enhance lung repair following VILI. Chimenti *et al.* demonstrated the potential for MSC pretreatment to prevent the development of VILI following high-volume ventilation. MSC pretreatment reduced the lung water content and improved the lung histology score ^19^. Bronchoalveolar lavage (BAL) levels of protein, neutrophils, macrophage inflammatory protein-2, and IL-1β were also considerably reduced ^7^. Curley *et al.* observed that intra-tracheal and intravenously delivered rat MSCs and their secretome comparably enhanced restoration of lung oxygenation and compliance and restored lung structure after high-pressure VILI ^20^. Inflammation was also significantly attenuated by MSC delivery as indicated by lower concentrations of IL-6 and TNF-α in the BAL ^20^. More recently, Hayes *et al.* showed that MSCs retained their therapeutic efficacy following high-pressure VILI at doses as low as 2 million cells per kilogram ( Table 1) ^21^. They also observed that efficacy with MSC treatment was maintained with delayed delivery (that is, 6 hours after injury) ^21^.

MSCs enhance alveolar fluid clearance, an important process in the recovery following ARDS. Bacteria-induced inflammation was also significantly inhibited and this appeared to be due to the enhanced phagocytic ability of macrophages and to antibacterial effects of secreted KGF in an *ex vivo* perfused human lung pneumonia model ^22^. Of importance in transplant medicine, MSCs also restored alveolar fluid clearance in another study which used *ex vivo* lungs that were ischemic and rejected for transplantation ( Table 1) ^23^.

## Strategies to enhance mesenchymal stromal/stem cell efficacy

A key early study demonstrated the potential to enhance MSC efficacy by using gene overexpression strategies, specifically overexpression of Ang-1 ^6^. More recently, MSCs overexpressing angiotensin-converting enzyme 2 (ACE2) showed enhanced endothelial repair and enhanced reduction in the expression of inflammatory molecules (including TNF-α and IL-6) following *in vitro* LPS injury when compared with naïve MSCs ^24^ ( Table 1). ACE2 plays a major role in negating the pro-inflammatory and pro-apoptotic effects of angiotensin II on the endothelium ^24^. *In vivo*, Min *et al.* showed that MSCs overexpressing ACE2 caused an enhanced reduction in inflammation and reduced lung edema, collagen deposition, and fibrosis after bleomycin-induced lung injury in mice ^25^. Furthermore, these cells decreased oxidative stress damage to a greater degree than naïve MSCs ^25^. Finally, human MSCs overexpressing soluble IL-1 receptor-like-1, which plays an immune-modulatory role in the lung, decreased inflammation, BAL protein and neutrophil content to a greater extent than naïve MSCs in a mouse model of LPS-induced ALI ^26^.

## Recent insights regarding the mesenchymal stromal/stem cell secretome

MSCs exert their therapeutic efficacy in part via paracrine mechanisms ( Table 2). MSC-CM—termed the ‘MSC secretome’—attenuates injury following endotoxin-induced ALI in the mouse ^27^, restoring lung structure and decreasing alveolar concentrations of IL-6 and macrophage inflammatory protein-2. These effects were partly mediated via the attenuation of the expression of nuclear factor-kappa B (NF-κB) p65 and phospho-NF-κB p65 in the mouse lung ^27^. MSC-CM also induced neutrophil apoptosis as indicated by a reduction of the expression of Bcl-xl and Mcl-1 on said neutrophils ^27^. In a similar model, MSC-CM enhanced the activation of the M2 macrophage phenotype, thus promoting an anti-inflammatory and pro-healing environment ^28^. Interestingly, these effects were correlated with increased levels of insulin-like growth factor 1 (IGF-1) ^18^.

**Table 2.** The mesenchymal stromal/stem cell secretome: recent preclinical studies.

Injury setting	Therapy	Effects
**Entire MSC secretome**		
Endotoxin-induced ALI in mice ^27^	Mouse MSC conditioned medium	- Decreased histology score and lung neutrophils - Decreased lung interleukin-6, macrophage inflammatory protein-2, and NF-κB p65 and phospho-NF-κB p65 expression - Increased apoptosis of neutrophils
**MSC-derived microvesicles**		
Endotoxin-induced ALI in mice ^30^	Human MSC microvesicles 30 μL	- Decreased lung edema - Decreased protein, neutrophil, and macrophage inflammatory protein-2 levels - In part via enhanced KGF expression
*Escherichia coli* pneumonia in mice ^32^	Human MSC microvesicles	- Increased survival - Decreased bacterial load - Decreased BAL protein - Increased phagocytosis by monocytes *in vitro*
*E. coli* pneumonia in mice ^33^	Human bone marrow MSCs	MSCs through mitochondrial transfer in microvesicles enhanced the phagocytic action of macrophages *in vitro* and *in vivo*

Abbreviations: ALI, acute lung injury; MSC, mesenchymal stromal/stem cell; NF-κB, nuclear factor-kappa B; KGF, keratinocyte growth factor; BAL, bronchoalveolar lavage.

The potential for the MSC secretome to enhance lung repair following VILI has also been demonstrated. Intra-tracheal delivery of the MSC secretome products enhanced lung oxygenation and compliance and reduced the lung histology score and pulmonary edema at 48 hours after high-pressure VILI ^20^. Another study examined the effects of MSCs and their secretome in the earlier phases of the repair process following VILI ^29^. The MSCs were effective and significantly improved blood oxygenation and respiratory compliance while reducing lung edema, BAL neutrophil counts, and IL-6 concentrations ^29^. In contrast, the MSC secretome failed to exert therapeutic effects at this earlier stage in the recovery process, suggesting that not all of the effects of the MSC are recapitulated by the secretome alone ^29^.

## Recent insights regarding mesenchymal stromal/stem cell-derived microvesicles and exosomes

A relatively new discovery is that some of the effects of MSCs appear to be mediated via the release of particles, specifically microvesicles and exosomes that can transfer genetic material, cellular organelles such as mitochondria, and biologically active molecules between cells ( Table 2). One study observed that microvesicles from hBM-MSCs significantly decreased lung edema and BAL protein and neutrophil counts in an LPS lung injury model and this was facilitated partly by the increased expression of KGF ^30^. Islam *et al.* demonstrated that MSCs delivered to LPS-injured mice transferred mitochondria-containing microvesicles through gap junctions to the mouse alveolar epithelia and that the therapeutic effect was abrogated with the use of MSCs with non-functional mitochondria or gap junction-incompetent MSCs ^31^. Another study observed that human MSC-derived microvesicles enhanced survival in a mouse model of *E. coli* pneumonia ^32^. The microvesicles were comparable to MSC cell treatment in their ability to reduce bacterial load and BAL protein and cytokine concentrations, and their effects were mediated in part by KGF secretion ^32^. Furthermore, the phagocytic capability of monocytes *in vitro* was also increased by the microvesicles ^32^. Jackson *et al.* also observed that MSCs enhanced macrophage phagocytosis *in vitro* and produced anti-bacterial effects in an *E. coli* pneumonia mouse model in part via microvesicle transfer of mitochondria ^33^.

## Clinical studies

Based on their therapeutic promise in preclinical studies, MSCs have entered early phase clinical testing in patients with ARDS ( Table 3). The recently reported phase 1b START trial (safety proof-of-concept trial) enrolled nine patients to receive 1, 5, or 10 million cells per kilogram of a single intravenous dose of allogeneic hBM MSCs using a three-by-three dose escalation design and showed no adverse effects at any of the doses used ^34^. These investigators are now enrolling patients in a phase 2 study using the dose of 10 million cells per kilogram (NCT01775774). In another phase 1 trial, adipose-derived allogeneic MSCs were administered to 12 patients in an intravenous dose of 1 million cells per kilogram or placebo in a 1:1 ratio and again the cells were well tolerated in the patients ^35^. This study also measured IL-6, IL-8, and surfactant protein D levels and found that surfactant protein D concentrations were lower after MSC delivery at day 5 but that MSC administration had no effects on IL-6 or IL-8 levels ^35^. The study concluded that higher doses may be needed at the phase 2 stage ^35^. Importantly, given the relationship between ARDS and sepsis, MSCs are also in early phase clinical studies in patients with septic shock in Canada (Cellular Immunotherapy in Septic Shock study, NCT02421484).

**Table 3.** Mesenchymal stromal/stem cell safety in clinical studies.

Study type	Cell therapy	Findings
Phase 1 clinical trial ^34^	Human bone marrow mesenchymal stromal/stem cells (MSCs) 1, 5, or 10 million cells per kilogram	No adverse effects detected
Phase 1 clinical trial ^35^	Human adipose MSCs 1 million per kilogram	Well tolerated

## Mesenchymal stromal/stem cells for acute respiratory distress syndrome: challenges and next steps

Although MSCs demonstrate considerable promise and early phase clinical trials are encouraging, several challenges still impede the clinical translation of MSCs for patients with ARDS. Cell batch variability between donors remains a significant hurdle that raises efficacy and possible safety concerns. Robust assays of MSC batch potency for ARDS are lacking. However, one recent study observed that cell motility can predict the differentiation potential of MSCs, which may provide a bioassay for characterizing MSC efficacy for certain disease conditions ^36^. Another study also recently outlined that MSCs from young mouse donors were more effective than those from aging donors in reducing pulmonary fibrosis ^37^. Of relevance to ARDS, MSCs from aging mice were less effective in reducing endotoxemia-induced lung injury ^38^. Importantly, the efficacy of these cells in an *in vitro* cell motility assay paralleled their effectiveness in subsequent *in vivo* studies, suggesting that this may be a useful assay of potency ^38^.

Furthermore, the generation of large quantities of clinical-grade MSCs for dose delivery to humans is both time consuming and costly. Doses of up to 1 billion MSCs per patient are currently being studied in clinical trials. Should MSCs prove an effective therapy at these doses, alternative, more easily accessible and abundant sources, such as adipose tissue or the umbilical cord, are likely to prove more feasible than bone marrow, which is the current source of cells used in most clinical studies. The demonstration that MSCs retain their efficacy when cryopreserved is important in making bulk production and storage of MSCs for clinical use feasible ^17^ and will facilitate MSC translation to the clinic.

In conclusion, MSCs may provide the immunomodulatory and regenerative tool needed to target ARDS pathogenesis. Preclinical studies have highlighted their therapeutic potential and mechanisms of action. These preclinical studies may also be of relevance to other clinical situations, such as smoke inhalation injury, lung transplantation, and chemotherapy-induced lung injury. Although there are considerable grounds for optimism regarding the therapeutic potential of MSCs for ARDS, hurdles to translation still exist, and only time will tell whether MSCs realize their therapeutic potential in later-phase clinical trials.

## Abbreviations

ACE2, angiotensin-converting enzyme 2; *ALI*, *acute lung injury;* ARDS, acute respiratory distress syndrome; *BAL*, *bronchoalveolar lavage;* hBM, human bone marrow; IL, interleukin; KGF, keratinocyte growth factor; LPS, lipopolysaccharide; MSC, mesenchymal stromal/stem cell; MSC-CM, mesenchymal stromal/stem cell conditioned medium; NF-κB, nuclear factor-kappa B; TNF-α, tumor necrosis factor-alpha; VILI, ventilator-induced lung injury.
